# Validation of a weight bearing ankle equinus value in older adults with diabetes

**DOI:** 10.1186/s13047-018-0306-x

**Published:** 2018-11-21

**Authors:** A. Searle MOsteo, M. J. Spink, V. H. Chuter

**Affiliations:** 1 School of Health Sciences, Faculty of Health, University of Newcastle, PO Box 127, Ourimbah, NSW 2258 Australia; 2 Priority Research Centre for Physical Activity and Nutrition, University of Newcastle, PO Box 127, Ourimbah, NSW 2258 Australia

**Keywords:** Dorsiflexion, Ankle, Diabetes, Equinus, Lunge

## Abstract

**Background:**

Accurate measurement of ankle joint dorsiflexion is clinically important as a restriction has been linked to many foot and ankle pathologies, as well as increased ulcer risk and delayed ulcer healing in people with diabetes. Use of the reliable weight bearing (WB) Lunge test is limited as normal and restricted ranges for WB ankle joint dorsiflexion are not identified. Additionally the extent of WB dorsiflexion restriction that results in clinically adverse outcomes is unclear. Therefore the aim of this investigation is to validate a proposed weight bearing equinus value (dorsiflexion < 30°) in unimpaired cohorts, and secondly to investigate any clinical effects this degree of ankle dorsiflexion restriction has on forefoot plantar pressure variables in older adults with diabetes.

**Methods:**

Ankle dorsiflexion was measured using a Lunge test with the knee extended in young adults without diabetes (YA) and older adults with diabetes (DA). In-shoe and barefoot plantar pressure was recorded for the DA group. Spearman’s correlation was calculated to determine any association between the presence of ankle equinus and plantar pressure variables in the DA group. DA group differences in people with and without an equinus were examined.

**Results:**

A weight bearing equinus of < 30°, assessed in a lunge using an inclinometer placed on the anterior tibia, falls within the restricted range in young unimpaired cohorts. In the DA group this degree of ankle restriction had a fair and significant association with elevated barefoot forefoot peak pressure (*r* = 0.274, *p* = 0.005) and pressure-time integrals (*r* = 0.321, *p* = .001). The DA equinus group had significantly higher barefoot peak pressure (mean kPa (SD): 787.1 (246.7) vs 652.0 (304.5), *p* = 0.025) and pressure-time integrals (mean kPa (SD): 97.8 (41.6) vs 80.4 (30.5), *p* = 0.017) than the DA non equinus group.

**Conclusions:**

We support a preliminary weight bearing ankle equinus value of < 30°. This value represents a restricted range in young adults and is correlated with increased forefoot plantar pressure variables in older adults with diabetes. Mean population weight bearing ankle dorsiflexion data presented here for older adults with diabetes, will allow use of the more functional Lunge test with knee extended in research and clinical practice.

## Introduction

Limited ankle dorsiflexion, ankle equinus, has been implicated in the development of many foot and ankle pathologies, including metatarsalgia, plantar fasciitis, high plantar pressures and the development and non-healing of plantar forefoot ulcer in people with diabetes [1–3]. The prevalence of ankle equinus is reported to be higher in people with diabetes, especially those with neuropathy, compared to people without diabetes [3]. A contributing factor is believed to be the non-enzymatic glycosylation of soft tissues seen with chronic hyperglycaemia, which results in structural changes in the Achilles tendon, increased tendon stiffness and reduced ankle joint mobility [4, 5]. The ankle dorsiflexion restriction acts to limit forward progression of the tibia over the foot during stance phase, leading to inadequate foot rollover, prolonged weight bearing at the forefoot, increased plantar pressures and increased risk of forefoot plantar ulcer [5, 6].

A reliable, relevant, easy to administer, clinic-based method of measuring ankle dorsiflexion is required for clinical decision making and research purposes. A review has identified ten different methods for measuring ankle dorsiflexion, including weight bearing (WB) and non-WB measurement, with knee extended and knee bent, and using specifically designed devices [7]. Despite known problems with reliability, non-WB measurement using a goniometer remains the most common method used for measuring ankle dorsiflexion [7]. In addition to reliability problems, the non-WB method may not be clinically relevant. Ankle dorsiflexion during WB and gait occurs in a closed kinetic chain and non-WB dorsiflexion is measured in an open chain. Low correlations have been shown between the two measures and authors have warned against clinical use of non-WB measurement for the last 25 years [8, 9]. When WB measurement has been used it has most commonly been measured with the knee flexed [7], removing the effects of the gastrocnemius muscle, which is believed to be the most prevalent cause of ankle dorsiflexion restriction [1].

In contrast, a WB Lunge test with the knee extended is reliable [10–12], more accurately reflects torque applied to the ankle during gait, and is clinically easy to administer using minimal equipment such as a goniometer, digital inclinometer or free smartphone applications that act as an inclinometer [12–14]. However, there are limited data available for comparison of the general population [8, 10, 11, 15, 16] or for populations with diabetes [17–19]. Additionally, while authors have variously defined a non-WB equinus as < 0 degrees, 0 degrees or less, < 5 degrees or < 10 degrees of ankle dorsiflexion [20], there is currently no recognised degree of WB ankle dorsiflexion restriction that indicates a restricted or pathological range. Baumbach et al. [15] have recently suggested that dorsiflexion less than 30 degrees, a value that fell below the 95% CI in the young healthy adult cohort they measured, should be regarded as restricted. This value has not been validated as a restricted range in other young adult populations, and the effects of this level of restriction have not been investigated in any populations where a dorsiflexion restriction may contribute to injury or pathology.

Consequently, the aim of this study was to validate that WB ankle dorsiflexion < 30 degrees does represent a WB dorsiflexion restricted range (a WB equinus), through examination of population mean values in a group of young unimpaired adults. Further, to investigate the clinical effects of this degree of WB ankle dorsiflexion restriction through any association with increased forefoot pressure variables in older people with diabetes.

## Methods

### Participants

Ethics approval was granted by the University of Newcastle Human Research Ethics Committee and written informed consent was obtained from all participants. Data from young adults without diabetes (YA), recruited between August and October 2016 from the student population of the University of Newcastle, Ourimbah, Australia, were used to determine unimpaired population mean WB ankle dorsiflexion values. Inclusion criteria for the YA group were adults, 45 years or under, without diabetes and without surgery to the foot or lower limb involving fixation of a joint. Older adults with diabetes (DA) were recruited from the University of Newcastle Podiatry Clinic at Wyong Hospital, NSW, Australia and from newspaper advertisements in local newspapers, between June 2016 and October 2017. Inclusion criteria for the DA group were adults, 65 years of age or older, able to provide informed consent and a diagnosis of either type 1 or type 2 diabetes for the diabetes group. Exclusion criteria for the DA group were existing foot ulcer affecting plantar pressure measurement, any previous lower limb amputation, any surgery to the foot or lower limb involving fixation of a joint, any neurological condition that may affect the lower limb other than loss of sensation due to diabetes, inability to walk 8 m unaided, or current pregnancy.

### Procedures

All data were collected at one testing session at the University of Newcastle Podiatry Clinic, Wyong Hospital. Testing was conducted on the participants’ dominant leg only to maintain independence of data [21]. Dominance was determined by asking the participant which foot they would kick a football with.

**Fig. 1:**
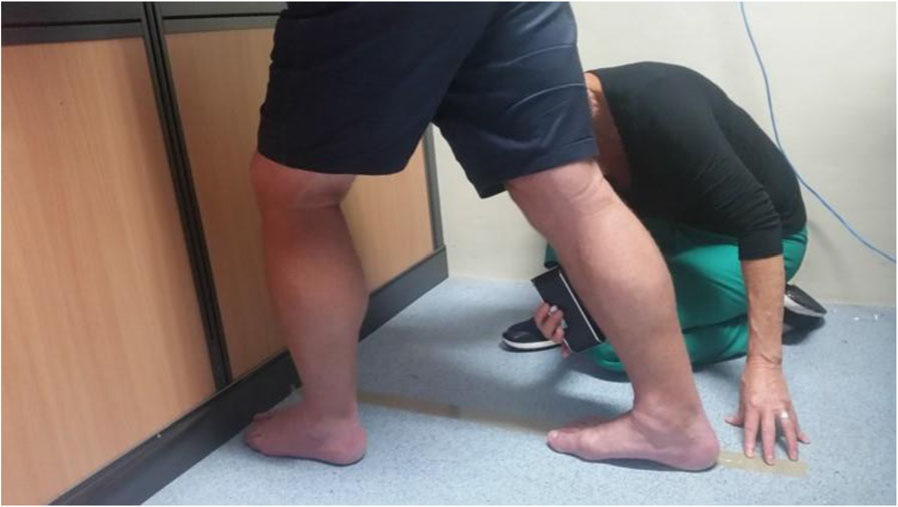
Measurement technique for the weight bearing Lunge test with knee extended using a digital inclinometer (Bear Digital Protractor 82201B-00, China)

A Lunge test with the knee extended and an inclinometer (Bear Digital Protractor 82201B-00, China) placed on the mid-point of the anterior border of the tibia, between the tibial tuberosity and the anterior joint line of the ankle, was used to determine WB ankle joint dorsiflexion range of motion (Fig. 1). The test was completed three times, with 10 s rest between tests, and the average score was documented as the test result. The authors have previously shown this method to have excellent intrarater (ICC = 0.83 to 0.89) and interrater reliability (ICC = 0.88 to 0.93), so a single assessor recorded all the measurements in this study [12]. In-shoe plantar pressures were measured with the Novel Pedar-X ® system (Novel GmbH, Munich, Germany) which utilises flexible insoles that contain 99 capacitive sensors sampling at a frequency of 100 Hz. Participants walked along a flat 12 metre walkway at their normal walking speed wearing an appropriately sized standardised shoe (New Balance® 624), with the insole placed between a standardised cotton sock and the shoe. The New Balance® 624 shoes are cushioned, neutral cross trainers weighing 355 g with a 1 cm heel pitch, and wide fittings were used in this trial. A minimum of two walking trials was required to capture twelve midgait dominant foot footsteps [22]. Barefoot plantar pressures were collected with the Tekscan HR Mat™ Pressure Measurement System (Tekscan Inc., South Boston, USA) using a 2-step protocol which has been shown to collect reliable pressure data [23]. The pressure platform contains 8448 individual pressure sensing cells sampling at 60 Hz, and participants were asked to look straight ahead while walking, to avoid targeting of the platform. The average of four successful trials was used for data analysis [23].

**Fig. 2:**
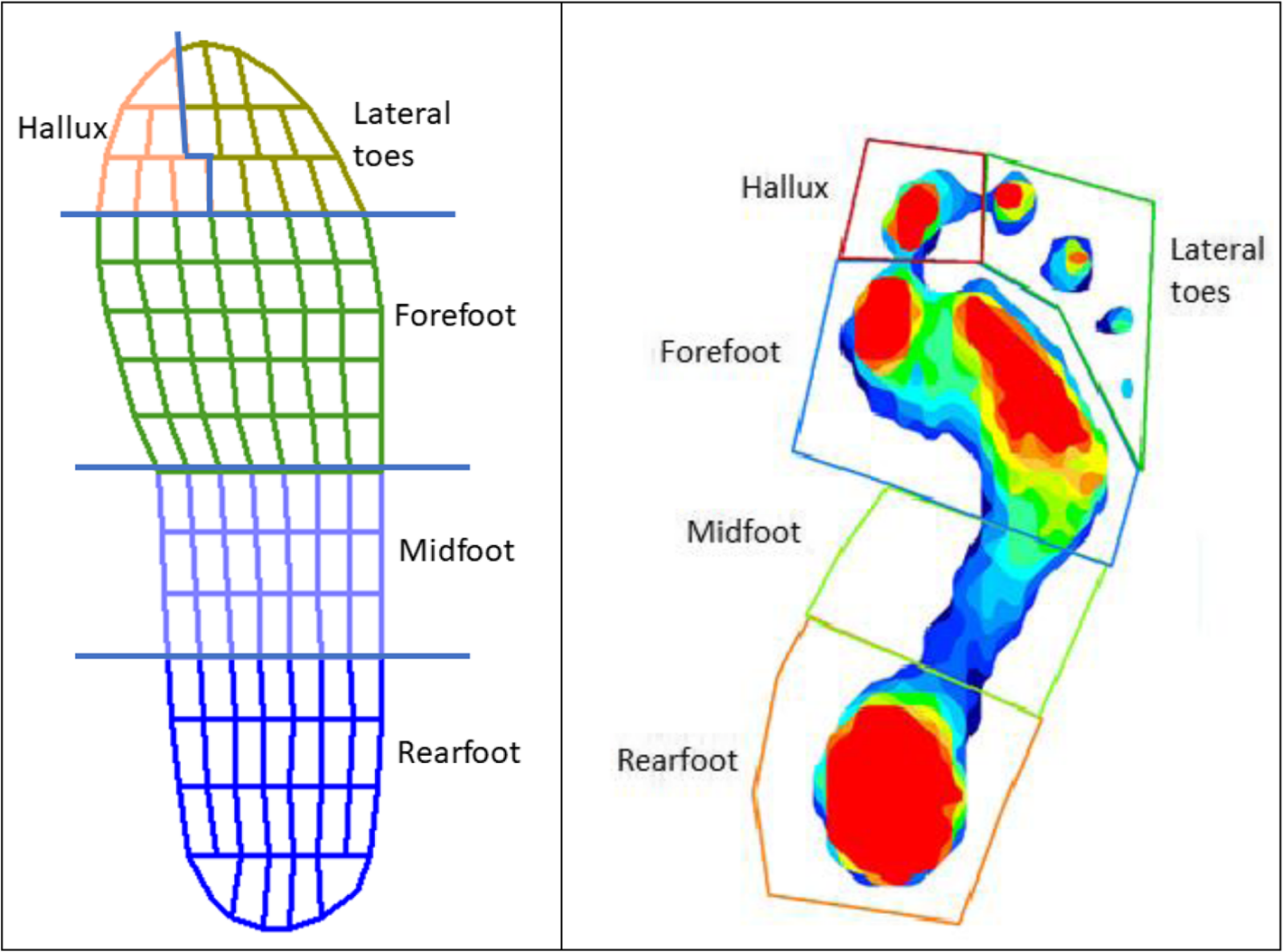
The five footprint masks (forefoot, midfoot, rearfoot, hallux and lateral toes) displayed for the Novel Pedar-X ® footprint at the left, and over a typical Tekscan HR Mat™ footprint on the right

For in-shoe and barefoot pressure measurement the foot was divided into five regions or masks: rearfoot, midfoot, forefoot, hallux and lateral toes (Fig. 2). Percentage masks were applied to each Pedar footprint, with the rearfoot and midfoot masks together occupying 50% of the total foot length, the forefoot the next 30%, the hallux occupying the medial 35% of the remaining foot length and the lateral toes occupying the rest [24]. HR Mat™ pressures were evaluated using a mask similar to that used in previous studies with the only change being consolidation of three metatarsophalangeal joint regions into one forefoot region [25]. Only the forefoot pressure variables are included in this statistical analysis as an ankle equinus is proposed to contribute to gait alterations that elevate forefoot plantar pressures and increase foot ulcer risk in at risk diabetes populations [26].

In the patients with diabetes, neuropathy status was assessed using a monofilament and a neurothesiometer which are reliable tests for measuring foot sensation [27, 28]. Four points on the plantar surface of the dominant foot (1st, 3rd and 5th metatarsal heads and the distal hallux) were tested with a 10 g Semmes-Weinsten monofilament. An abnormal test was noted if the participant failed to identify the monofilament at one or more test sites [29]. A neurothesiometer (Horwell, Bailey Instruments, Manchester, UK) was used to detect the vibration perception threshold (VPT) at the pulp of the hallux. Three readings were taken and the average used in analysis. A VPT value of > 25 V was regarded as abnormal [29]. Participants were assessed as neuropathic if they recorded one or more abnormal test results.

### Statistical analysis

To determine unimpaired population mean WB ankle dorsiflexion values, a conservative sample size of 40 participants in the YA group was calculated based on n = (Z * SD)^2^/d^2^, where Z = 1.96, SD (standard deviation) = 5.6 degrees (using SD values from Baumbach et al. [15]) and d (absolute error) = 2 degrees [30]. All statistical tests were conducted using SPSS Release 24 for Windows (SPSS Inc., Chicago, Ill., USA*)*. Mean, standard deviation (SD) and 95% confidence intervals (CI) were calculated for WB ankle dorsiflexion for YA and DA groups. DA participants were grouped into those with and those without a WB equinus (defined as WB ankle dorsiflexion < 30 degrees). Spearman rank coefficients were calculated to assess any correlation between the dichotomous measure of the presence of a WB equinus and forefoot plantar pressure variables (peak pressure and pressure time integrals) in the DA group. The correlation coefficients were interpreted as a small (0.1 to 0.25), fair (0.25 to 0.5), moderate (0.5 to 0.75) and strong (0.75 to 1.0) relationship [31]. Differences in plantar pressure variables between the equinus and non equinus groups were evaluated by independent samples t-test [31]. To determine the likelihood of high forefoot peak pressure (PP) occurring in those with WB equinus compared to those without, Chi square analysis was used to calculate the unadjusted odds ratio with 95% confidence intervals between WB equinus and non equinus and at risk barefoot peak pressure (PP) (> 700 kPa) and low PP (700 kPa or less) [32]. While no definitive peak pressure value that predicts ulceration has been determined, a level of 700 kPa has been shown to have optimal sensitivity and specificity for identifying groups with current or recently healed plantar ulcers. [32] Statistical significance was delimited at *P* < 0.05.

## Results

**Fig. 3:**
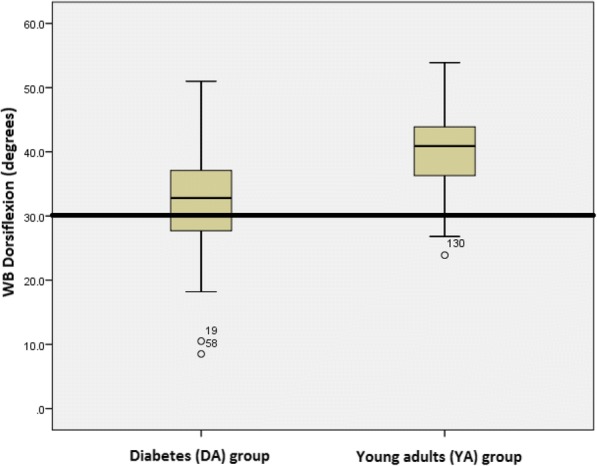
Boxplots of weight bearing (WB) ankle dorsiflexion (degrees), measured with the Lunge test with the knee extended, for the diabetes (DA) group on the left and the young adult (YA) group on the right. End horizontal bars represent minimum and maximum dorsiflexion values (excepting outliers) and the middle horizontal bars represent the median values. Line at 30 degrees indicates level of weight bearing equinus

One hundred and four adults were recruited to the DA group, and forty adults to the YA group (Table 1). The majority of the DA participants had a diagnosis of Type 2 diabetes (95.2%) and 3 people (2.9%) reported a history of foot ulcer. Ankle dorsiflexion measured with the Lunge test with the knee extended for DA and YA groups was normally distributed, with minimal outliers exerting no influence on the reported mean values. The DA group displayed reduced ankle dorsiflexion motion (95% CI 30.8° to 33.6°) compared to the YA group (95% CI 38.0° to 42.5°) (Fig. 3).

**Table 1:** Characteristics of the study population

	Diabetes (*n* = 104)	Diabetes equinus (*n* = 35)	Diabetes non equinus (*n* = 69)	Young Adults (*n* = 40)
Age (years)	73.3 (5.6)	73.8 (5.8)	73.1 (5.5)	28.8 (7.9)
Male (n)	54 (52.0%)	12 (34.3%)	42 (60.9%)	16 (40%)
BMI (kg/m^2^)	32.6 (5.9)	33.4 (5.8)	32.3 (6.0)	25.7 (5.9)
Duration of diabetes (years)	15.5 (11.6)	18.1 (13.5)	14.1 (10.3)	0
Neuropathy	54 (51.9%)	15 (42.8%)	39 (56.5%)	0
HbA1c (*n* = 81)	7.1 (1.2)	7.2 (1.0)	7.1 (1.2)	–

Values are mean (SD) unless stated otherwise

Values for mean and standard deviation WB ankle dorsiflexion for the YA group and other young adult comparison groups without diabetes are reported in Table 2. WB ankle dorsiflexion of less than 30 degrees, measured at the anterior tibia, can be seen to fall in the restricted (hypomobile) range in the YA group in this study. Four (10%) of the YA cohort displayed this degree of restriction. Values for mean and standard deviation WB ankle dorsiflexion for the DA group and other diabetes comparison groups are also reported in Table 2. The smaller dorsiflexion means reported in some diabetes comparison groups may be explained by the populations examined. High risk diabetes populations, such as those classified at high risk to develop foot ulcer [19], or with a history of Charcot’s neuroarthropathy or sensory neuropathy [17], may be expected to display more diabetes related complications, including limited joint mobility, than the community dwelling low risk DA group.

In the DA group, there was a fair and significant association between the presence of a WB equinus, defined as weight bearing ankle dorsiflexion less than 30 degrees, and elevated barefoot forefoot PP (*r* = 0.274, *p* = 0.005) and PTIs (*r* = 0.321, *p* = .001), but no association with in-shoe plantar pressure variables. When DA participants were grouped into those with and those without a WB equinus (Table 3), significantly higher values were found in the equinus group compared to the non equinus group for barefoot forefoot PP (mean kPa (SD): 787.1 (246.7) vs 652.0 (304.5), *p* = 0.025) and PTIs (mean kPa/s (SD): 97.8 (41.6) vs 80.4 (30.5), *p* = 0.017). People in the DA group with a WB equinus were two and a half times as likely to present with at risk barefoot PP (> 700 kPa) [32] as those without equinus (odds ratio 2.5, 95% CI: 1.1 to 5.7, *p* = < 0.025).

**Table 2:** Mean and 95% confidence interval and normative values (degrees) for weight bearing ankle dorsiflexion with knee extended

Author	No. people (feet)	Measure method	Mean (SD)	95% CI	Excessive hypo mobility (<−2 SD)	Hypo-mobility (− 2 to −1 SD)	Normal (−/+ 1 SD)	Hyper-mobility (+ 1 to + 2 SD)	Excessive hyper mobility (> + 2 SD)
Diabetes groups									
DA group	104 (104)	AT	32.2 (7.2)	30.8–33.6	<=17.7	17.8–24.9	25.0–39.4	39.5–46.6	> = 46.7
Raspovic^a^ [18]	30 (30)	AT	35.0 (6.1)^f^		<=22.7	22.8 to 28.8	28.9 to 41.1	41.2 to 47.2	> = 47.3
Chuter^a^ [17]	41 (82)	LL	15.6 (5.4)^f^		<=4.9	5.0 to 10.2	10.3 to 21.0	21.1 to 26.3	> = 26.4
Tang^a^ [19]	74 (148)	AT	26.5 (7) ^e^		<=12.5	12.6 to 19.4	19.5 to 33.5	33.6 to 40.4	> = 40.5
Non-diabetes groups									
YA group	40 (40)	AT	40.2 (7.0)	38.0–42.5	<=26.2	26.3–33.1	33.2–47.2	47.3–54.1	> = 54.2
Munteanu^a^ [10]	30 (30)	AT	39.0 (4.6)^d^		<=29.8	29.9–34.3	34.4–43.6	43.7–48.1	> = 48.2
Baumbach^a^ [15]	64 (128)	LL	33.6 (5.6)^e^	31.9–34.7 (L) 32.1–35.0 (R)	<=22.5	22.6–27.9	28.0–39.2	39.3–44.6	> = 44.7
Krause^a^ [11]	39 (39)	LL	33.2 (7.2)^d^		<=18.8	18.9–25.9	26.0–40.4	40.5–47.5	> = 47.6
Konrad^a^ [16]	38 (38)	LL	31.5 (6.6)^f^		<=18.2	18.3–24.8	24.9–38.1	38.2–44.6	> = 44.7
Baggett^b^ [8] Baggett^c^	30 (60)	LL	20.9 (6.8)		<=7.3	7.4–14.1	7.0–34.7 14.2–27.9	28.0–34.7	> = 34.8

*DA* Diabetes group, *YA* Young adults group, ^a^normative values calculated from grouped mean data available in article using our defined reference range calculations, ^b^stated values from article (within 2 SDs of the mean), ^c^values calculated from data available in article using our defined reference range calculations, ^d^calculated from grouped mean rater data, ^e^averaged from left and right feet, ^f^averaged from group data, *AT* anterior tibia, *LL* lateral leg, *L* left, *R* right

**Table 3:** Mean (SD) forefoot plantar pressure variables for the diabetes (DA) group, grouped by equinus and non-equinus (where equinus is ankle dorsiflexion < 30 degrees)

Plantar Pressure Variable	Total population (*n* = 104)	Equinus (*n* = 35)	Non Equinus (*n* = 69)	*p* value
Barefoot Peak Pressure (kPa)	697.5 (292.2)	787.1 (246.7)	652.0 (304.4)	0.025^*^
Barefoot PTI (kPa/s)	86.3 (35.4)	97.8 (41.6)	80.4 (30.5)	0.017^*^
In-shoe Peak Pressure (kPa)	233.3 (53.7)	239.6 (54.4)	230.1 (53.5)	0.400
In-shoe PTI (kPa/s)	83.0 (23.9)	85.3 (24.1)	81.8 (23.8)	0.484

*PTI* pressure time integral, ^*^significant difference between groups

## Discussion

This study first sought to validate a recent proposal that WB ankle dorsiflexion of less than 30 degrees, measured with the Lunge test with knee extended, represents a WB ankle restriction in unimpaired cohorts. Following that, to investigate if this degree of WB ankle dorsiflexion restriction has any clinical implications, by examination of any effects on plantar pressures in older adults with diabetes. Our results show that WB ankle dorsiflexion less than 30 degrees, when measured at the anterior tibia, does fall in a restricted range in a young healthy cohort, and is also associated with elevated barefoot forefoot plantar pressures in older adults with diabetes.

Due to lack of a gold standard definition for a WB equinus, historically authors have used a number of methods to classify the condition. These have included subjective clinical assessment of impairment, using values two SDs below the mean of the reference group, and values derived from dynamic gait assessment [33–35]. However, subjective assessment is no longer considered valid [33], and the values from the gait assessment trials show such a wide variation that they are not clinically useful [34, 35]. Statistically only 2.5% of the population falls two SDs below the mean, so while use of this method may capture extreme ankle dorsiflexion hypomobility, it would not identify smaller and possibly clinically relevant ankle dorsiflexion restrictions. A new method, recently suggested by Baumbach et al. is that dorsiflexion values below the 95% CI in a young healthy adult cohort should be regarded as restricted, and they propose a value of WB ankle dorsiflexion of less than 30 degrees [15].

There are only a limited number of studies in young adult cohorts available for comparison with the mean WB dorsiflexion results reported by Baumbach et al. [15] (Table 2). Comparable results are reported by two other trials using the same measurement technique, where a goniometer is placed on landmarks on the lateral leg [11, 16]. Much smaller mean WB dorsiflexion values are reported by another trial measuring with a wedge placed under the foot to limit sub talar joint motion [8]. This method could be expected to result in smaller mean dorsiflexion values than measurement methods that do not control for movement of the subtalar and more distal joints as these are likely to contribute additional sagittal plane movement. Larger mean WB ankle dorsiflexion values are reported in the YA group from this present study and by Munteanu et al. [10] Both of these trials used a slightly different measurement technique to that of Baumbach et al. [15], with placement of a goniometer on the anterior tibia. Rabin et al. [36] have reported that ankle dorsiflexion values measured using the Lunge test with an inclinometer placed at the anterior tibia, are 5 to 8 degrees higher than when measured with an inclinometer placed at the lateral leg. While ankle dorsiflexion was measured with a flexed knee in that trial, it is credible that similar higher values would result from measurement with an extended knee. The figures reported by Munteanu et al. [10] and the YA group from this study are comparable to the other studies, including that of Baumbach et al. [15], when a 5 degree measurement difference is taken into account.

If the normative ranges for WB ankle dorsiflexion for the comparison studies are examined (Table 2) then the proposed restricted value of 30 degrees of dorsiflexion is seen to fall within the normal (−/+ 1 SD) range for Baumbach et al. and the other two studies where dorsiflexion was measured with the inclinometer placed on the lateral leg [11, 15, 16]. A smaller value than that suggested by Baumbach et al. [15], that falls in the hypomobile range (− 2 to − 1 SD) of the cohorts, may better represent a restricted value. A value of less than 25 degrees of WB ankle dorsiflexion, when measured at the lateral leg, is within the hypomobile range of these studies (Table 2). If the 5 to 8 degrees measurement difference when the inclinometer is placed on anterior tibia is taken into account, this would be equivalent to a value of less than 30 degrees. For the two studies (the YA group and the Muntenau et al. [10] study) where dorsiflexion was measured with the inclinometer placed on the anterior tibia this value of less than 30 degrees also falls in hypomobile range (− 2 to − 1 SD).

The second aim was to investigate the clinical effects of a WB ankle equinus in the DA group. A WB equinus value of 30 degrees was used in these calculations because, as described above, we believe this value to be representative of an ankle dorsiflexion restriction when measured at the anterior tibia. An ankle equinus is proposed to restrict the forward progression of the tibia over the foot during stance phase, resulting in gait alterations that elevate forefoot plantar pressures thereby increasing foot ulcer risk in at risk diabetes populations [26]. In support of this theory we have demonstrated that a WB equinus has significant effects on barefoot forefoot plantar pressure variables in older adults with diabetes. In the DA group we found a fair and significant association between presence of a WB equinus (ankle dorsiflexion < 30 degrees measured at the anterior tibia) and elevated barefoot forefoot PP (*r* = 0.274, *p* = 0.005) and PTIs (*r* = 0.321, *p* = .001). When the DA participants were grouped as WB equinus or non equinus, barefoot forefoot PP and PTIs were significantly higher in the equinus group (Table 3). Equinus group PTIs, which are 16.6% higher than the non equinus group, are indicative of a longer forefoot stance time. Both a shift in weight bearing from rearfoot to forefoot and an early heel lift have been seen in people with limited ankle dorsiflexion [35, 37], and could result in early and prolonged forefoot loading and higher PTIs. Furthermore, in the equinus group only, mean barefoot forefoot PP was above the 700 kPa level suggested by Armstrong et.al [32] as a risk level for ulceration. Calculation of odds ratios revealed that DA group participants with a WB ankle equinus were two and a half times as likely to present with these at risk barefoot PPs as those without an equinus.

In the DA group no association was found between the presence of a WB ankle equinus and in-shoe pressure variables, and no between group differences were noted for in-shoe pressure variables, which may be explained by the pressure relieving effects of the shoes. Running shoes similar to those used in our trial have been shown to result in lower plantar pressures compared to barefoot in people with diabetes and may result from a greater foot contact area in the shoes [38].

These results suggest that WB ankle dorsiflexion less than 30 degrees, measured with the WB Lunge test with knee extended at the anterior tibia, does have deleterious effects for people with diabetes, through elevation of barefoot plantar pressures. Previous studies have indicated that people with diabetes with high barefoot pressures are three to four times more likely to develop foot ulcer than those with low pressures [39]. Assessment of WB ankle dorsiflexion restriction, easily performed in clinical situations using the WB Lunge test with the knee extended, would help identify people with diabetes at risk of increased plantar pressures, and associated increased risk of foot ulcer. The lack of findings for in-shoe pressure variables supports the guidelines for people with diabetes to wear protective footwear [39]. As the standard running shoes used in this trial appear to have alleviated any effects the ankle dorsiflexion restriction had on elevating plantar pressures, similar shoes could be recommended to low risk diabetes cohorts found to have a WB equinus.

Our results should be viewed in light of several limitations. No attempt was made to control for subtalar joint pronation except for placement of the participants foot on the tape line on the floor. It is probable that a combination of talocrural, subtalar and tarsal joint motion contributed to the overall range of motion recorded. However this is a more accurate indication of actual motion occurring at the ankle complex during activities of daily living. Only a limited number of studies are available for comparison with the mean WB ankle dorsiflexion data that the proposed WB ankle equinus value is based on, and further studies may result in changes to this value. The WB equinus value was only investigated with regard to plantar pressures in older people with diabetes, and it is unknown if this level of restriction will also be correlated with conditions traditionally associated with limited ankle dorsiflexion, such as metatarsalgia, plantar fasciitis, Achilles tendonitis and risk of plantar foot ulcer in people with diabetes.

## Conclusions

We support a definition for a WB ankle equinus, measured with the WB Lunge test at the anterior tibia with the knee extended, of dorsiflexion less than 30 degrees. This value is associated with increased barefoot forefoot plantar pressure variables in people with diabetes. The normative ranges described for WB ankle dorsiflexion for older adults with diabetes (95% CI 30.8° to 33.6°) and young adults (95% CI 38.0° to 42.5°) may improve the utility of the WB Lunge test and allow clinicians and researchers to more easily identify a WB dorsiflexion restriction.

## Abbreviations

CI: Confidence intervals ICC Intra class coefficient
DA: Older adults with diabetes
PP: Peak pressure
PTI: Pressure time integrals
SD: Standard deviation
WB: Weight bearing
YA: Young adults without diabetes
